# Association between wide-ranging food intake and Parkinson’s disease: a comprehensive mendelian randomization study

**DOI:** 10.1038/s41598-025-85668-x

**Published:** 2025-01-18

**Authors:** Yana Su, Yulei Hao, Wanhui Dong, Ruqing Qiu, Ying Zhang

**Affiliations:** Present Address: Department of Neurology and Neuroscience Center, The First Hospital of Jilin University, Jilin University, 1 Xinmin Street, Changchun City, Jilin Province China

**Keywords:** Parkinson’s disease, Food intake, Mendelian randomization, Dietary habits, Parkinson's disease, Nutrition

## Abstract

Parkinson’s disease (PD) is a complex neurodegenerative disorder influenced by both genetic and environmental factors, including dietary habits. Despite considerable research, the relationship between food intake and PD risk remains poorly understood. Here, we conducted a comprehensive Mendelian randomization analysis to investigate the association between a wide spectrum of food intake and PD risk. Utilizing data from large-scale genome-wide association studies (GWAS) and dietary databases, we constructed genetic instruments for various dietary factors, including fruit, vegetable, meat, fish, dairy, and grain intake, among others, totaling 170 different food items. Using multivariable inverse variance weighted methods, we found a causal relationship between Mozzarella intake and Parkinson’s disease (odds ratio [OR] = 9.83, 95% confidence interval [CI] = 2.52–38.34, P-value < 0.05). Additionally, a causal relationship was observed between Pancake intake and Parkinson’s disease (odds ratio [OR] = 0.20, 95% confidence interval [CI] = 0.07–0.59, P-value < 0.05). Furthermore, our reverse Mendelian randomization analysis and multivariable Mendelian randomization analysis provided further support for these findings. To our knowledge, we are the first to investigate the causal relationship between the broad intake of 170 different food items and Parkinson’s disease. Our study reveals the causal relationships between Pancake intake, and Mozzarella intake with Parkinson’s disease.

**Supplementary Information:**

The online version contains supplementary material available at 10.1038/s41598-025-85668-x.

## Introduction

Parkinson’s disease (PD) is the second most common neurodegenerative disease after Alzheimer’s disease (AD) characterized by degeneration and loss of dopaminergic neurons in the substantia nigra pars compacta(SNpc)^1^. According to The Global Burden of Disease Study, there were over 6 million PD cases worldwide in 2015, and that number will double to 12 million cases by 2040 ^2^. Currently, there is no preventative or curative therapy for PD. It seriously affects the daily life of patients and increases the burden on society as disease progresses. Although the etiology of PD is not fully understood, it is generally believed that both genetic and environmental factors contribute to its development^3^. Among the latter, there has been growing interest in the impact of dietary intake on the development of PD.

Previous studies have investigated the relationship between dietary habits and PD. Many dietary factors have been established to be associated with PD risk. Observational studies found that the diet abundant in vegetables, fruits, and fish with low intake of dairy products reduced the risk of PD^4,5^. Additionally, the Mediterranean diet and the MIND diet have also been linked to a reduced risk of PD^6–8^. A few studies have also identified some food risk factors for PD progression. In a cross-sectional study, Mischley et al. found that increased intake of fresh vegetables, fresh fruit, nuts and seeds, fish, olive oil, wine, coconut oil, fresh herbs were significantly associated with the reduction in PD severity. It was found that the consumption of canned fruits, vegetables, diet and non-diet sodas, fried foods, beef, ice cream, yogurt, and cheese were associated with the aggravation of PD^9^. Epidemiological studies have shown an association between food intake and PD. However, it is unclear whether this association is causal due to potential reverse causation or confounding factors. Therefore, further exploration is needed to understand the association between the two.

Mendelian randomization (MR) is an emerging method that employs genetic variation as instrumental variables (IVs) for risk factors to evaluate causal relationships between exposures and outcomes^10^. MR analysis is based on three core assumptions: (i) the exposure is strongly associated with the IVs, (ii) IVs are not related to confounding factors for exposures and outcomes, and (iii) genetic variants affect outcome only through exposure^11^. The MR approach is less susceptible to potential reverse causation or confounding factors which may distort interpretations of conventional observational studies^12,13^. Therefore, it can be particularly beneficial to examine the potential long-term consequences of risk factors. In comparison to traditional observational clinical studies, MR has unique advantages over other research methods when examining the influence of dietary factors on disease. At present, MR has been widely used to elucidate the causal relationship between dietary factors and disease. Furthermore, the MR approach is less susceptible to potential reverse causation or confounding factors^12^. However, there are few MR studies on the causal relationships between food intake and PD. Therefore, the objective of this research was to use MR based on two-sample summary data to analyze the causal relationship between a wide spectrum of food intake and PD risk. Our study may provide new insights into targeted dietary interventions to prevent PD.

## Methods

### Study design

**Fig. 1:**
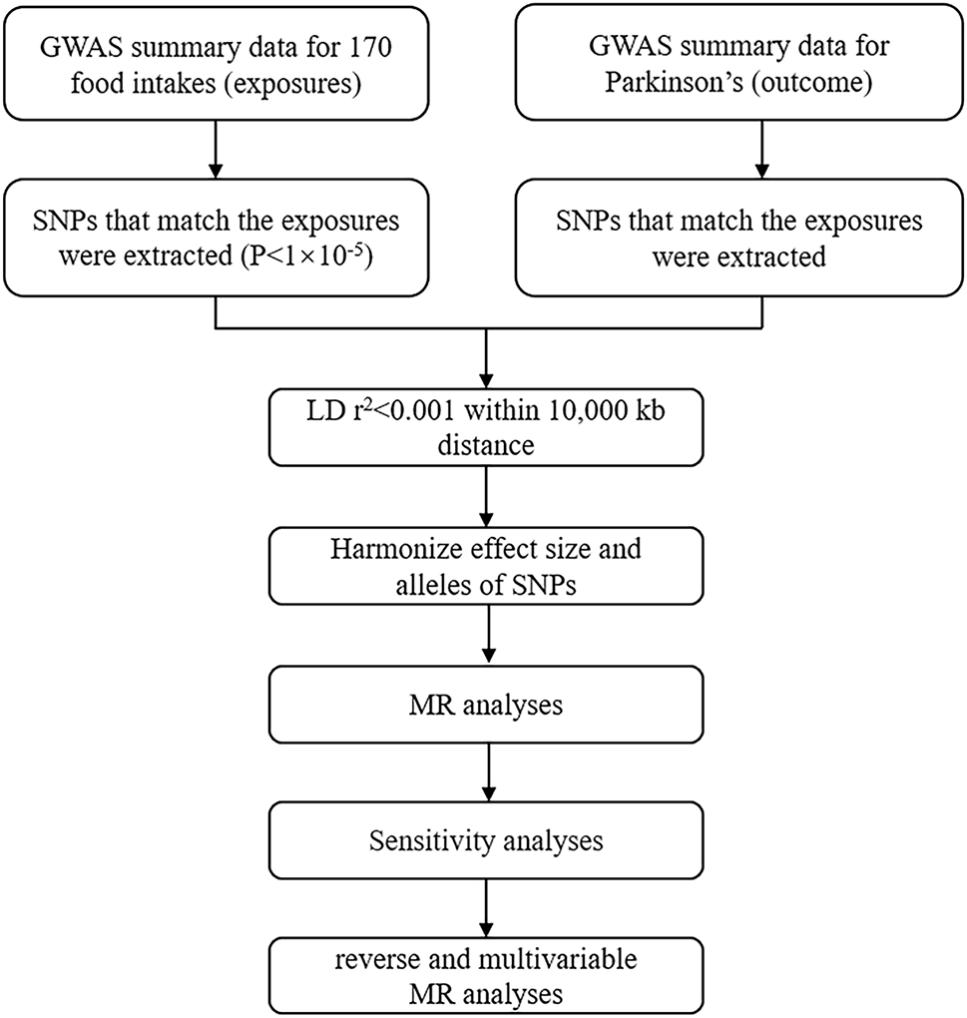
Flowchart of MR analysis in this study.

This study followed the guidelines of the STROBE-MR statement^14,15^. In this study, food intake was used as exposure variables and single nucleotide polymorphisms (SNPs) loci significantly associated with them were selected as IVs, and the outcome variable was PD. This study employed a two-sample MR approach to investigate the causal relationship between a wide array of factors related to diet exposure and PD risk (Fig. 1). MR needs to satisfy three fundamental assumptions. The first assumption is that genetic variation was significantly associated with food intake. The second was that genetic variants were not associated with any confounders of the exposure-outcome association. The last assumption was that genetic variants affect PD risk only through food intake.

## Food intake GWAS data

Summary statistics for food intake are all available from the IEU Open GWAS project (https://gwas.mrcieu.ac.uk/datasets/), which primarily comprises publicly available GWAS summary data. We selected 170 food intake phenotypes as exposure factors, including fruit, vegetable, meat, fish, dairy, and grain intake, among others. More information about the exposure datasets is presented in Supplementary Table S1. The significance level for each instrumental variable (IV) for food intake phenotypes was set at 1 × 10^(-5). We pruned these single nucleotide polymorphisms (SNPs) (within a linkage disequilibrium [LD] r^2^ threshold of < 0.001 within 10,000 kb distance) (Fig. 1).

## PD GWAS data

The GWAS data pertaining to PD has been obtained from the GWAS catalog. With ebi-a-GCST90018894, we extracted the variable genetic information of PD from the IEU Open GWAS project (https://gwas.mrcieu.ac.uk/datasets/), which is a publicly available GWAS summary database. Each instrumental variable (IV) was considered significant at a threshold of 1 × 10^ (-5). We pruned the SNPs using a threshold of < 0.001 for linkage disequilibrium (LD) within a distance of 10,000 kb (Fig. 1).

## Data sources and processing

The study’s data were sourced from the MRC IEU Open GWAS project (https://gwas.mrcieu.ac.uk/datasets/), an open GWAS dataset platform managed by the University of Bristol. This resource provides access to over 2 million quality-controlled GWAS summary statistics, ensuring their accuracy and scientific validity. The datasets used comply with the platform’s open data terms of use.

During data processing, we adhered to the platform’s data usage and quality control standards. All datasets underwent a standardized quality control process to ensure result reliability. We also complied with relevant ethical requirements throughout the data analysis and interpretation to ensure legal and standardized data use.

## Tools and methods

This study employed the Two-Sample MR tool for two-sample Mendelian randomization analyses. Two-Sample MR is an open-source R package developed by the MRC Integrative Epidemiology Unit (MRC IEU), with source code and usage guidelines available on GitHub (https://github.com/MRCIEU/TwoSampleMR). It is designed to assess causal relationships between exposure factors and outcomes across different samples and is widely employed in causal inference studies utilizing publicly available GWAS datasets.

Two-Sample MR offers several features: it includes a built-in interface for accessing a wide array of GWAS data, enabling easy retrieval from public resources. Additionally, it provides various methods for quality control, effect estimation, and sensitivity analyses to ensure result accuracy and reliability. Furthermore, Two-Sample MR supports multiple techniques, such as inverse variance weighting, weighted median, and weighted mode, allowing researchers to validate result robustness under different analytical conditions.

### Ethics statement

Our research relied on publicly accessible GWAS summary statistics data sourced from the European Biobank. Comprehensive integration of gene expression data was conducted. All data collection procedures were conducted in compliance with the approval of respective institutional review boards, and informed consent was obtained from all study participants. This study did not necessitate individual ethical approval.

### Statistical analysis

Our MR analysis predominantly relied on the inverse variance-weighted (IVW) method, implemented through the R package Two-Sample MR. To address potential horizontal pleiotropy, we applied the MR-Egger method, a widely recognized technique that identifies significant horizontal pleiotropy by examining its intercept term. We assessed the heterogeneity in SNP-specific causal effects using Cochran’s Q-test during the two-sample MR analysis. Additionally, we conducted a leave-one-out sensitivity analysis to evaluate the impact of individual SNPs on the overall estimates, considering heterogeneity present when the P-value for Cochran’s Q-statistic was below 0.05.

Furthermore, we employed reverse Mendelian randomization and multivariable Mendelian randomization to further support our findings. Reverse Mendelian randomization (MR) and multivariable Mendelian randomization offer several advantages in validating causal relationships inferred from traditional MR analyses. Firstly, reverse Mendelian randomization allows researchers to investigate the causal effects of a specific exposure on multiple outcomes. By leveraging genetic instruments for the exposure of interest and conducting analyses across various outcome phenotypes, researchers can gain insights into the broader impact of the exposure on health outcomes. Secondly, multivariable Mendelian randomization enables the assessment of multiple exposures simultaneously while accounting for potential confounding factors. This approach helps disentangle complex relationships between exposures and outcomes, providing more robust and reliable causal estimates. Moreover, both reverse and multivariable Mendelian randomization techniques offer increased statistical power and improved control over confounding compared to traditional observational studies. By leveraging genetic variants as instrumental variables, these methods mitigate biases arising from confounding and reverse causation inherent in observational data, leading to more reliable causal inference. Additionally, the use of genetic instruments ensures that the assumptions of Mendelian randomization are more likely to be met, enhancing the validity of causal inference. This strengthens the credibility of findings and facilitates the translation of research into clinical and public health interventions. Overall, the incorporation of reverse Mendelian randomization and multivariable Mendelian randomization in causal inference studies enhances the robustness, validity, and generalizability of research findings, thereby advancing our understanding of complex relationships between exposures and outcomes. The bioinformatics analysis was conducted using the R software alongside various bioinformatics tools.

## Results

### The discussion of the causal effect between 170 different food intake and PD

**Fig. 2:**
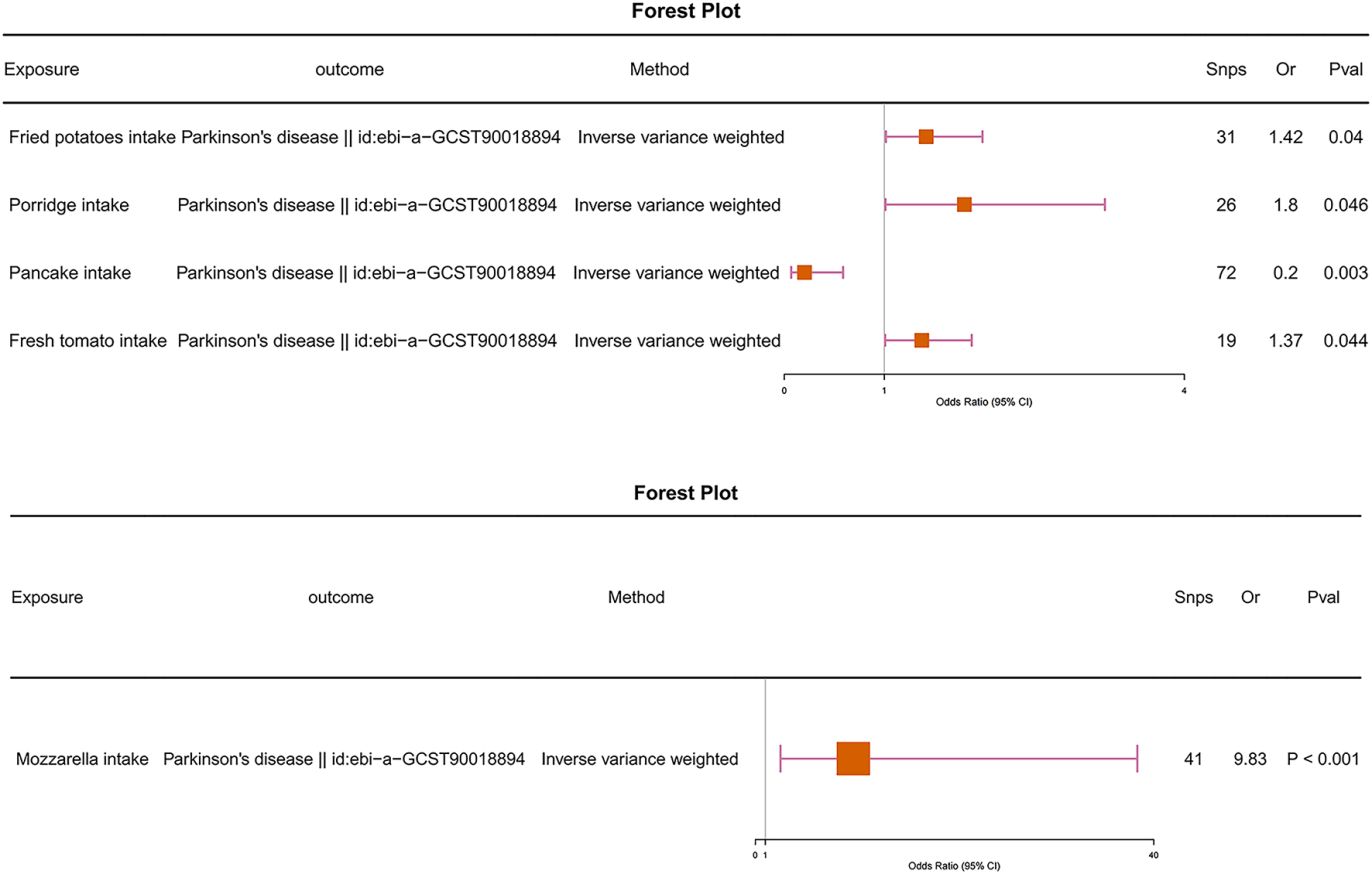
The causal relationship between food intake and PD was analyzed using the IVW method.

We conducted a univariable Mendelian randomization analysis to examine the potential causal relationship between PD and 170 different food intake variables. Employing primarily the IVW method, our findings unveiled significant causal associations between PD and several dietary factors. Specifically, Fried potatoes intake showed a positive correlation with PD (odds ratio [OR] = 1.42, 95% confidence interval [CI] = 1.02–1.98, P-value < 0.05), as did Mozzarella intake (odds ratio [OR] = 9.83, 95% confidence interval [CI] = 2.52–38.34, P-value < 0.05), and Pancake intake displayed a negative correlation (odds ratio [OR] = 0.20, 95% confidence interval [CI] = 0.07–0.59, P-value < 0.05). Additionally, our analysis indicated causal relationships between PD and Porridge intake, as well as Fresh tomato intake (Fig. 2).

We ensured the credibility of our findings through thorough assessments for horizontal pleiotropy, heterogeneity, and Leave-One-Out (LOO) analysis. Horizontal pleiotropy was examined using the MR-Egger method. Across all five causal relationships, the P-values for the MR-Egger regression intercept exceeded 0.05, indicating the absence of heterogeneity in our analysis results (see Supplementary Table S2 online). LOO analysis consistently depicted trends across all included SNPs, with scatter plots further confirming the robustness of our results (see Supplementary Fig. S1, S2 online). Heterogeneity tests resulted in p-values exceeding 0.05, suggesting homogeneity in our analysis outcomes. Thus, the reliability of our findings remains upheld (see Supplementary Table S3 online).

### Validation through reverse mendelian randomization and multivariable mendelian randomization

**Fig. 3:**
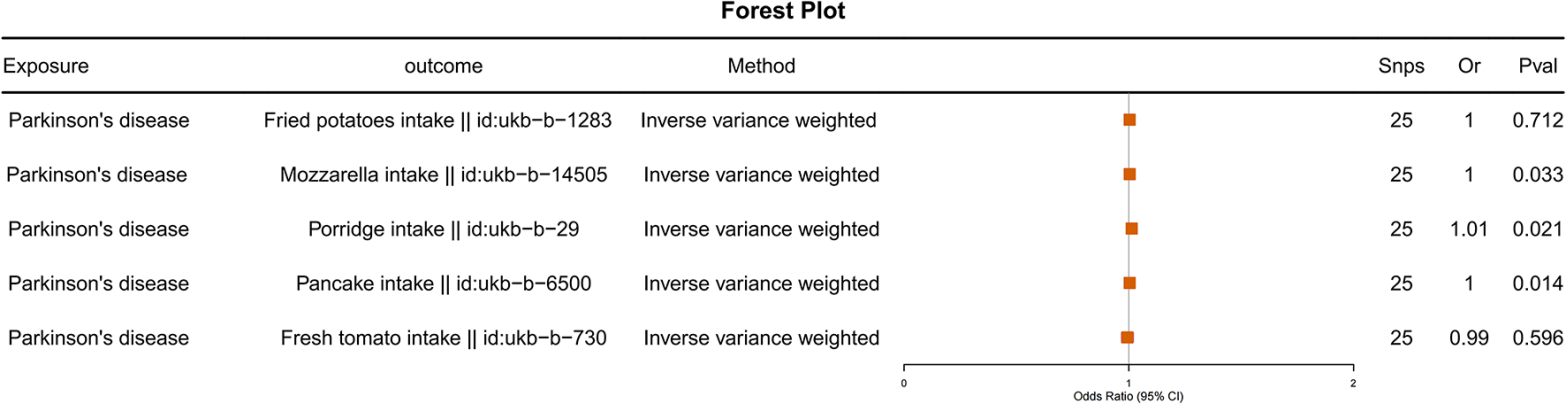
The causal relationship between PD as exposure and food intake was analyzed using reverse Mendelian randomization.

**Fig. 4:**
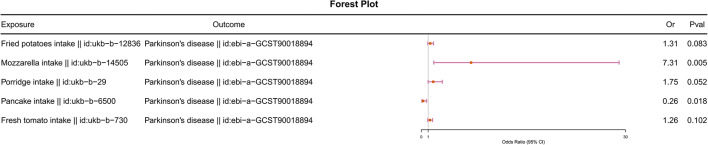
The causal relationship between PD and five types of food intake was analyzed using multivariable Mendelian randomization.

To further validate our findings, we conducted reverse Mendelian randomization analyses with PD as the exposure and five food intakes as outcomes. The results indicated no causal relationship between PD and any of the five food intakes (odds ratio [OR] = 1, p-values > 0.05) (Fig. 3). Additionally, we performed multivariable Mendelian randomization analyses with the five food intakes as exposures and PD as the outcome. After adjusting for mutual effects, Fried potatoes intake, Porridge intake and Fresh tomato intake showed no causal relationship with PD (p-values > 0.05). However, Mozzarella intake remained associated with increased risk of PD, while Pancake intake remained protective against PD (Fig. 4).

## Discussion

To the best of our knowledge, this study conducted extensive MR analyses to investigate the causal relationship between various food intakes and PD. We identified a causal link between two food intakes and PD: mozzarella may increase the risk, while pancakes may reduce the risk.

Although the relationship between dairy products and PD has been investigated previously, the conclusion is inconsistent. Several prospective studies found a positive association between dairy food intake and PD incidence^16,17^, while a cohort study in Greece found that milk consumption was positively linked with a higher risk of PD, but not cheese or yogurt consumption^18^. Another cohort study found there was no significant association between total dairy intake and PD risk, but low-fat dairy intake was associated with an increased risk of PD^19^. Consumption of ice cream, cheese, and yogurt was associated with higher rates of PD progression^9^. However, a case-control study found no association between dairy intake and PD^20^. Most of the publications have studied the effects of dairy intake in combination, and it is still unclear which single type of dairy intake is associated with PD. Our study only found that Mozzarella intake is positively associated with the risk of PD, but did not find a significant association between other types of dairy intake and PD risk. Different dairy products vary widely in their nutrient and compound content, which may explain the observed differences. Mozzarella is a fresh, fine-textured Italian cheese made from the milk of buffalo. Currently, there is a lack of research on the relationship between Mozzarella intake and PD, and the underlying mechanisms the causal correlation between the two are unknown. Mozzarella is a variety of dairy product, there may be several potential mechanisms to explain the causal relationship between dairy intake and the risk of PD. Intake of dairy products reduces uric acid^21^. Low levels of uric acid are associated with an increased risk of PD and more rapid progression of the disease^22–25^. Another potential explanation is that dairy products may contain neurotoxic ingredients or contaminants, such as pesticides^26,27^. Further studies may be required in the future to validate these results and in depth the potential mechanisms that exist.

In addition, our findings also indicated that higher pancake intake may be a protective factor for PD. There is a lack of studies on the relationship between pancake consumption and PD. Most of studies only focused on broad food categories rather than specific foods. Further research is needed to validate our MR findings. Previous studies reported a significant association between the consumption of potatoes and tomatoes and the risk of PD^28,29^, however, this association does not exist after adjusting for mutual effects in our MR analysis. Several other foods have been linked to PD risk in previous studies. Higher intakes fresh fruits and vegetables, nuts, fish, and coffee are protective against PD^5,7,30–34^, while consumption of red meat may increase the risk of PD^35,36^. However, our MR analysis did not find any association between these food groups intake and the risk of PD. Further clinical trials are needed to explore the potential impact of diet on PD.

The main strength of the study is the MR analysis design, which reduces potential reverse causation or confounding factors, thereby improving the causal inference of the associations^12^. The findings of this study bring new insights and implications for the treatment and dietary choices of PD patients. Firstly, we identified specific food intake associated with the risk of PD, providing guidance for patients to make wiser choices in their daily diet. For instance, understanding the association between mozzarella consumption and an increased risk of PD may encourage patients to reduce or avoid consuming this food. On the contrary, we found that Pancake intake may be associated with a reduced risk of PD, providing valuable clues for preventive measures. Additionally, for patients undergoing PD treatment, understanding the relationship between specific food intake and disease progression may help optimize treatment plans. Adjusting the diet could potentially alleviate symptoms or slow down disease progression.

However, our study also has several limitations worth noting. Firstly, the GWAS data used in our analysis originated solely from European databases, limiting the generalizability of our findings to populations of other ethnicities. Considering different susceptibility in various region, further studies are needed to validate our findings in different populations and countries and to strengthen the significance of the results. Secondly, we focus exclusively on food type, overlooking potentially relevant other factors such as food quantity. Future studies are planned to incorporate detailed data on dietary habits, such as quantity and frequency of food intake, providing a more comprehensive understanding of the role of dietary intake in PD. Thirdly, it is possible that an increase in the size of the GWAS dataset might introduce SNPs with significant small effects, which in turn could affect the robustness of the results. Further research is required to verify the findings in larger and more diverse samples. Additionally, using GWAS datasets for Mendelian Randomization (MR) studies can introduce potential biases, particularly due to population stratification and the assumptions inherent in MR analyses. A more rigorous approach to handling these biases in future studies could enhance the reliability of causal inferences in dietary research. Lastly, although our MR study provided some genetic evidence for the causal relationship between food intake and PD, our study focused mainly on a methodological level, highlighting the need for extensive longitudinal cohort studies and long-term controlled trials to thoroughly evaluate the potential of diet in PD prevention and to reveal the detailed biological mechanisms underlying these relationships.

## Conclusions

Overall, this study highlights the potential impact of dietary factors on the development and progression of PD, providing new insights into its prevention and management. These findings may contribute to future research and the design of personalized therapeutic strategies.

## Electronic supplementary material

Below is the link to the electronic supplementary material.

Supplementary Material 1

Supplementary Material 2

Supplementary Material 3

## Data Availability

Our research relied on openly accessible data sets. The genome-wide association study (GWAS) summary statistics were obtained from the IEU Open GWAS Project (https://gwas. mrcieu.ac.uk/datasets/). The GWAS ID in Supplementary Table S1 can be entered in the website to query and download the GWAS dataset used in this article.

## Abbreviations

AD: Alzheimer’s disease
CI: Confidence interval
GWAS: Genome-wide association studies
IVs: Instrumental variables
IVW: Inverse variance-weighted
LD: Linkage disequilibrium
LOO: Leave-One-Out
MR: Mendelian randomization
OR: Odds ratio
PD: Parkinson’s disease
SNpc: Substantia nigra pars compacta
SNPs: Single nucleotide polymorphisms
