# Supplementary material for: Association between wide-ranging food intake and Parkinson’s disease: a comprehensive mendelian randomization study
Source: Sci Rep. 2025 Jan 18;15:2374. doi: 10.1038/s41598-025-85668-x (PMC11742879; doi:10.1038/s41598-025-85668-x)
Supplement: Supplementary file 3 — Supplementary Material 3 [file 41598_2025_85668_MOESM3_ESM.docx]

**STROBE-MR checklist of recommended items to address in reports of Mendelian randomization studies**^1^ ^2^

| **Item No.** | **Section** | **Checklist item** | **Page No.** | **Relevant text from manuscript** |
| --- | --- | --- | --- | --- |
| 1 | **TITLE and ABSTRACT** | Indicate Mendelian randomization (MR) as the study’s design in the title and/or the abstract if that is a main purpose of the study | 1 | Association between Wide-ranging Food Intake and Parkinson's Disease: A Comprehensive Mendelian Randomization Study |
|  | **INTRODUCTION** |  |  |  |
| 2 | **Background** | Explain the scientific background and rationale for the reported study. What is the exposure? Is a potential causal relationship between exposure and outcome plausible? Justify why MR is a helpful method to address the study question | 3-4 | Parkinson's disease is a complex neurodegenerative disorder influenced by both genetic and environmental factors, including dietary habits.  Epidemiologic studies have shown an association between food intake and PD.  The main reason for its advantage in inferring causality is that MR employs the genetic variants as instrumental variables. |
| 3 | **Objectives** | State specific objectives clearly, including pre-specified causal hypotheses (if any). State that MR is a method that, under specific assumptions, intends to estimate causal effects | 4 | The objective of this research was to use MR based on two-sample summary data to analyze the causal relationship between a wide spectrum of food intake and PD risk. Our study may provide new insights into targeted dietary interventions to prevent PD.  Mendelian randomization (MR) is an emerging method that employs genetic variation as instrumental variables (IVs) for risk factors to evaluate causal relationships between exposures and outcomes. The MR approach is less susceptible to potential reverse causation or confounding factors. |
|  | **METHODS** |  |  |  |
| 4 | **Study design and data sources** | Present key elements of the study design early in the article. Consider including a table listing sources of data for all phases of the study. For each data source contributing to the analysis, describe the following: |  |  |
|  | a) | Setting: Describe the study design and the underlying population, if possible. Describe the setting, locations, and relevant dates, including periods of recruitment, exposure, follow-up, and data collection, when available. | 4-5 and Supplementary Table S1 | Detailed information, such as recruitment criteria of population and quality control of genetic data, can be found in the original paper (Supplementary Table S1 ). |
|  | b) | Participants: Give the eligibility criteria, and the sources and methods of selection of participants. Report the sample size, and whether any power or sample size calculations were carried out prior to the main analysis | 4-5 and Supplementary Table S1 | Detailed information, such as recruitment criteria of population and quality control of genetic data, can be found in the original paper (Supplementary Table S1) |
|  | c) | Describe measurement, quality control and selection of genetic variants | 4-5 and Supplementary Table S1 | Detailed information, such as recruitment criteria of population and quality control of genetic data, can be found in the original paper (Supplementary Table S1) |
|  | d) | For each exposure, outcome, and other relevant variables, describe methods of assessment and diagnostic criteria for diseases | 4-5 and Supplementary Table S1 | Detailed information, such as recruitment criteria of population and quality control of genetic data, can be found in the original paper (Supplementary Table S1) |
|  | e) | Provide details of ethics committee approval and participant informed consent, if relevant | 5 | Our research relied on publicly accessible GWAS summary statistics data sourced from the European Biobank. All data collection procedures were conducted in compliance with the approval of respective institutional review boards, and informed consent was obtained from all study participants. This study did not necessitate individual ethical approval. |
| 5 | **Assumptions** | Explicitly state the three core IV assumptions for the main analysis (relevance, independence and exclusion restriction) as well assumptions for any additional or sensitivity analysis | 4-5 and Figure1 | Presented in mendelian randomization analysis section of the Methods and Figure 1. |
| 6 | **Statistical methods: main analysis** | Describe statistical methods and statistics used |  |  |
|  | a) | Describe how quantitative variables were handled in the analyses (i.e., scale, units, model) | NA |  |
|  | b) | Describe how genetic variants were handled in the analyses and, if applicable, how their weights were selected | 4-5 | The significance level for each instrumental variable (IV) for food intake phenotypes was set at 1×10^(-5). We pruned these single nucleotide polymorphisms (SNPs) (within a linkage disequilibrium [LD] r^2 threshold of <0.001 within 10,000 kb distance). Each instrumental variable (IV) was considered significant at a threshold of 1×10^(-5). We pruned the SNPs using a threshold of <0.001 for linkage disequilibrium (LD) within a distance of 10,000 kb. |
|  | c) | Describe the MR estimator (e.g. two-stage least squares, Wald ratio) and related statistics. Detail the included covariates and, in case of two-sample MR, whether the same covariate set was used for adjustment in the two samples | 5 | IVW |
|  | d) | Explain how missing data were addressed | NA |  |
|  | e) | If applicable, indicate how multiple testing was addressed | NA |  |
| 7 | **Assessment of assumptions** | Describe any methods or prior knowledge used to assess the assumptions or justify their validity | 6 | Overall, the incorporation of reverse Mendelian randomization and multivariable Mendelian randomization in causal inference studies enhances the robustness, validity, and generalizability of research findings, thereby advancing our understanding of complex relationships between exposures and outcomes. |
| 8 | **Sensitivity analyses and additional analyses** | Describe any sensitivity analyses or additional analyses performed (e.g. comparison of effect estimates from different approaches, independent replication, bias analytic techniques, validation of instruments, simulations) | 7-8 | We ensured the credibility of our findings through thorough assessments for horizontal pleiotropy, heterogeneity, and Leave-One-Out (LOO) analysis. Horizontal pleiotropy was examined using the MR-Egger method. Across all five causal relationships, the P-values for the MR-Egger regression intercept exceeded 0.05, indicating the absence of heterogeneity in our analysis results (see Supplementary Table S2 online). LOO analysis consistently depicted trends across all included SNPs, with scatter plots further confirming the robustness of our results (see Supplementary Fig. S1, S2 online). Heterogeneity tests resulted in p-values exceeding 0.05, suggesting homogeneity in our analysis outcomes. Thus, the reliability of our findings remains upheld (see Supplementary Table S3). |
| 9 | **Software and pre-registration** |  |  |  |
|  | a) | Name statistical software and package(s), including version and settings used |  | MR were performed using the “Two Sample MR” (version 0.5.6) and R (version 4.3.1), |
|  | b) | State whether the study protocol and details were pre-registered (as well as when and where) | NA | This is a secondary analysis based on summary statistics from existing, published studies. The ethical approval and informed consent have been obtained by all original studies. |
|  | **RESULTS** |  |  |  |
| 10 | **Descriptive data** |  |  |  |
|  | a) | Report the numbers of individuals at each stage of included studies and reasons for exclusion. Consider use of a flow diagram | No Applicable |  |
|  | b) | Report summary statistics for phenotypic exposure(s), outcome(s), and other relevant variables (e.g. means, SDs, proportions) | 4-5 | The summary statistics for each food intake phenotype can be publicly accessed from the GWAS catalog (login credentials have been aggregated in the table) (Table S1). This includes 170 food intake phenotypes, such as fruit, vegetable, meat, fish, dairy, and grain intake, among others. The significance level for each instrumental variable (IV) for food intake phenotypes was set at 1×10^(-5). The GWAS data pertaining to PD has been obtained from the GWAS catalog (login credentials: ebi-a-GCST90018894).  Details can be found in the original studies. |
|  | c) | If the data sources include meta-analyses of previous studies, provide the assessments of heterogeneity across these studies | No Applicable |  |
|  | d) | For two-sample MR:  i.  Provide justification of the similarity of the genetic variant-exposure associations between the exposure and outcome samples  ii.  Provide information on the number of individuals who overlap between the exposure and outcome studies |  | These GWAS sample populations needed to be predominantly of European descent and largely independent of each other. |
| 11 | **Main results** |  |  |  |
|  | a) | Report the associations between genetic variant and exposure, and between genetic variant and outcome, preferably on an interpretable scale | 7, Figure2 | Mozzarella intake showed a positive correlation with PD (odds ratio [OR] = 9.83, 95% confidence interval [CI] = 2.52-38.34, P-value < 0.05), and Pancake intake displayed a negative correlation (odds ratio [OR] = 0.20, 95% confidence interval [CI] = 0.07-0.59, P-value < 0.05). |
|  | b) | Report MR estimates of the relationship between exposure and outcome, and the measures of uncertainty from the MR analysis, on an interpretable scale, such as odds ratio or relative risk per SD difference | 7, Figure2 | Mozzarella intake showed a positive correlation with PD (odds ratio [OR] = 9.83, 95% confidence interval [CI] = 2.52-38.34, P-value < 0.05), and Pancake intake displayed a negative correlation (odds ratio [OR] = 0.20, 95% confidence interval [CI] = 0.07-0.59, P-value < 0.05). |
|  | c) | If relevant, consider translating estimates of relative risk into absolute risk for a meaningful time period | No Applicable |  |
|  | d) | Consider plots to visualize results (e.g. forest plot, scatterplot of associations between genetic variants and outcome versus between genetic variants and exposure) | 8, Supplementary Fig. S1, S2 | LOO analysis consistently depicted trends across all included SNPs, with scatter plots further confirming the robustness of our results (see Supplementary Fig. S1, S2 online). |
| 12 | **Assessment of assumptions** |  |  |  |
|  | a) | Report the assessment of the validity of the assumptions | 5-8 | Methods to assess the robustness of MR findings: MR Egger, weighted median, Cochran's Q statistical test, and leave-one-out analyses. |
|  | b) | Report any additional statistics (e.g., assessments of heterogeneity across genetic variants, such as *I^2^*, Q statistic or E-value) | 8, Supplementary Table S3 | Heterogeneity tests resulted in p-values exceeding 0.05, suggesting homogeneity in our analysis outcomes. Thus, the reliability of our findings remains upheld (see Supplementary Table S3 online). |
| 13 | **Sensitivity analyses and additional analyses** |  |  |  |
|  | a) | Report any sensitivity analyses to assess the robustness of the main results to violations of the assumptions | 7-8, Supplementary Table S2,3 and Figure S1,2 | Supplementary Table S2,3 and  Figure S1,2 for the main analyses |
|  | b) | Report results from other sensitivity analyses or additional analyses | 7-8 | Presented in the Results. |
|  | c) | Report any assessment of direction of causal relationship (e.g., bidirectional MR) | 8 | To further validate our findings, we conducted reverse Mendelian randomization analyses with PD as the exposure and five food intakes as outcomes. The results indicated no causal relationship between PD and any of the five food intakes (odds ratio [OR] = 1, p-values > 0.05) (Fig. 3). |
|  | d) | When relevant, report and compare with estimates from non-MR analyses | NA |  |
|  | e) | Consider additional plots to visualize results (e.g., leave-one-out analyses) | 7-8, Supplementary Fig. S1, S2 | LOO analysis consistently depicted trends across all included SNPs, with scatter plots further confirming the robustness of our results (see Supplementary Fig. S1, S2 online) |
|  | **DISCUSSION** |  |  |  |
| 14 | **Key results** | Summarize key results with reference to study objectives | 8 | We identified causal relationships between two food intakes and PD, with the consumption of Mozzarella increasing risk of PD. On the contrary, our results suggest that pancake consumption may be protective against PD. |
| 15 | **Limitations** | Discuss limitations of the study, taking into account the validity of the IV assumptions, other sources of potential bias, and imprecision. Discuss both direction and magnitude of any potential bias and any efforts to address them | 11 | However, our study also has several limitations worth noting. Firstly, the GWAS data used in our analysis originated solely from European databases, limiting the generalizability of our findings to populations of other ethnicities. Further studies are required to validate our findings in different populations and countries in future research. Secondly, we considered only the types of foods, without taking into account other important factors such as the amount of food intake and the food interactions. Lastly, although our MR study provided some genetic evidence for the causal relationship between food intake and PD, further research is necessary to fully assess the potential of diet in PD prevention and to reveal the detailed biological mechanisms underlying these relationships. |
| 16 | **Interpretation** |  |  |  |
|  | a) | Meaning: Give a cautious overall interpretation of results in the context of their limitations and in comparison with other studies | 9-10 | Presented in the paragraph 2-3 of Discussion. |
|  | b) | Mechanism: Discuss underlying biological mechanisms that could drive a potential causal relationship between the investigated exposure and the outcome, and whether the gene-environment equivalence assumption is reasonable. Use causal language carefully, clarifying that IV estimates may provide causal effects only under certain assumptions | 9-10 | There may be several potential mechanisms to explain the causal relationship between dairy intake and the risk of PD. Intake of dairy products reduces uric acid. Low levels of uric acid are associated with an increased risk of PD and more rapid progression of the disease. Another potential explanation is that dairy products may contain neurotoxic ingredients or contaminants, such as pesticides. |
|  | c) | Clinical relevance: Discuss whether the results have clinical or public policy relevance, and to what extent they inform effect sizes of possible interventions | 11 | Overall, this study provides important guidance for the prevention and management of Parkinson's disease, emphasizing the role of dietary factors in the onset and progression of the disease, laying the groundwork for future research and the development of personalized treatment plans. |
| 17 | **Generalizability** | Discuss the generalizability of the study results (a) to other populations, (b) across other exposure periods/timings, and (c) across other levels of exposure | 11 | Firstly, the GWAS data used in our analysis originated solely from European databases, limiting the generalizability of our findings to populations of other ethnicities. Further studies are required to validate our findings in different populations and countries in future resear |
|  | **OTHER INFORMATION** |  |  |  |
| 18 | **Funding** | Describe sources of funding and the role of funders in the present study and, if applicable, sources of funding for the databases and original study or studies on which the present study is based | 14 | This study was supported by the Natural Science Foundation of Jilin Province (NO. YDZJ202201ZYTS116) to YZ, Jilin Province Department of Finance Project (NO. JLSWSRCZX2020-0011) to YZ and Jilin Province Health and Technology Innovation Project (NO.2018J045) to YZ |
| 19 | **Data and data sharing** | Provide the data used to perform all analyses or report where and how the data can be accessed, and reference these sources in the article. Provide the statistical code needed to reproduce the results in the article, or report whether the code is publicly accessible and if so, where | 14 | Availability of data and materials |
| 20 | **Conflicts of Interest** | All authors should declare all potential conflicts of interest | 14 | The authors declare that they have no conflicts of interest. |

This checklist is copyrighted by the Equator Network under the Creative Commons Attribution 3.0 Unported (CC BY 3.0) license.

1. Skrivankova VW, Richmond RC, Woolf BAR, Yarmolinsky J, Davies NM, Swanson SA, et al. Strengthening the Reporting of Observational Studies in Epidemiology using Mendelian Randomization (STROBE-MR) Statement. JAMA. 2021;under review.

2. Skrivankova VW, Richmond RC, Woolf BAR, Davies NM, Swanson SA, VanderWeele TJ, et al. Strengthening the Reporting of Observational Studies in Epidemiology using Mendelian Randomisation (STROBE-MR): Explanation and Elaboration. BMJ. 2021;375:n2233.
