# Supplementary material for: Association between wide-ranging food intake and Parkinson’s disease: a comprehensive mendelian randomization study
Source: Sci Rep. 2025 Jan 18;15:2374. doi: 10.1038/s41598-025-85668-x (PMC11742879; doi:10.1038/s41598-025-85668-x)
Supplement: Supplementary file 2 — Supplementary Material 2 [file 41598_2025_85668_MOESM2_ESM.docx]

**Supplementary file**

**The captions for supplementary materials**

Figure S1. Leave-one-out plot reveal the reliability of the causal relationship between foods intake and PD.

Figure S2. Scatter plots demonstrate the reliability of the causal association between foods intake and PD.

Table S2. Horizontal pleiotropy analysis reveals the reliability of the study results.

Table S3. Heterogeneity analysis reveals the reliability of the study results.

Figure S1. Leave-one-out plot reveal the reliability of the causal relationship between foods intake and PD. (a. Fried potatoes intake, b. Fresh tomato intake, c. Mozzarella intake, d. Porridge intake, e. Pancake intake)

Figure S2. Scatter plots demonstrate the reliability of the causal association between foods intake and PD. (a. Fried potatoes intake, b. Fresh tomato intake, c. Mozzarella intake, d. Porridge intake, e. Pancake intake)

**Table S2**. Horizontal pleiotropy analysis reveals the reliability of the study results.

| exposure | outcome | egger_ intercept | se | pval |
| --- | --- | --- | --- | --- |
| Fried potatoes intake  \|\| id: ukb-b-12836 | PD \|\| id: ebi-a-GCST90018894 | -0.01372 | 0.018523 | 0.464909 |
| Mozzarella intake  \|\| id: ukb-b-14505 | PD \|\| id: ebi-a-GCST90018894 | 0.004928 | 0.018339 | 0.789562 |
| Porridge intake  \|\| id: ukb-b-29 | PD \|\| id: ebi-a-GCST90018894 | 0.019104 | 0.022524 | 0.404719 |
| Pancake intake  \|\| id: ukb-b-6500 | PD \|\| id: ebi-a-GCST90018894 | -0.00857 | 0.013622 | 0.531212 |
| Fresh tomato intake  \|\| id: ukb-b-730 | PD \|\| id: ebi-a-GCST90018894 | 0.025943 | 0.022676 | 0.268455 |

**Table S3**. Heterogeneity analysis reveals the reliability of the study results.

| exposure | outcome | method | Q | Q_ df | Q_ pval |
| --- | --- | --- | --- | --- | --- |
| Fried potatoes intake  \|\| id: ukb-b-12836 | PD\|\| id: ebi-a-GCST90018894 | MR Egger | 23.63888608 | 29 | 0.746528984 |
|  |  | Inverse variance weighted | 24.18732766 | 30 | 0.76348371 |
| Mozzarella intake  \|\| id: ukb-b-14505 | PD\|\| id: ebi-a-GCST90018894 | MR Egger | 26.59437523 | 39 | 0.934674825 |
|  |  | Inverse variance weighted | 26.66658512 | 40 | 0.947564969 |
| Porridge intake  \|\| id: ukb-b-29 | PD\|\| id: ebi-a-GCST90018894 | MR Egger | 25.17474278 | 24 | 0.396315095 |
|  |  | Inverse variance weighted | 25.92936099 | 25 | 0.411376068 |
| Pancake intake  \|\| id: ukb-b-6500 | PD\|\| id: ebi-a-GCST90018894 | MR Egger | 71.42891475 | 70 | 0.430052636 |
|  |  | Inverse variance weighted | 71.83299648 | 71 | 0.450039909 |
| Fresh tomato intake  \|\| id: ukb-b-730 | PD\|\| id: ebi-a-GCST90018894 | MR Egger | 12.53514261 | 17 | 0.766706421 |
|  |  | Inverse variance weighted | 13.84400263 | 18 | 0.739202411 |
